# Lauder Brunton on Headaches

**Published:** 1894-07

**Authors:** 


LAUDER BRUNTON calculates that 80 to 90 per cent. of all headaches are due to defects of vision (hypermetropia, myopia, astigmatism, disparity in the focal distances of both eyes, deficient power of convergence). Ten per cent. are caused by bad teeth, and about 5 per cent. by affections of the nose, throat, ears or scalp, and other factors. The headache of the first group is usually seated in the forehead, the temples, or the occiput· where the visual power is not the same in both eyes, it frequently attacks the weaker side.

